# Radiation-resistant bacteria in desiccated soil and their potentiality in applied sciences

**DOI:** 10.3389/fmicb.2024.1348758

**Published:** 2024-06-04

**Authors:** Asaf Khan, Guangxiu Liu, Gaosen Zhang, Xiangkai Li

**Affiliations:** ^1^Ministry of Education Key Laboratory of Cell Activities and Stress Adaptations, School of Life Sciences, Lanzhou University, Lanzhou, China; ^2^Key Laboratory of Extreme Environmental Microbial Resources and Engineering, Lanzhou, China; ^3^Key Laboratory of Desert and Desertification, Northwest Institute of Eco-Environment and Resources, Chinese Academy of Sciences, Lanzhou, China

**Keywords:** desert, radiation, ROS species, antioxidant, environmental stresses, applications

## Abstract

A rich diversity of radiation-resistant (Rr) and desiccation-resistant (Dr) bacteria has been found in arid habitats of the world. Evidence from scientific research has linked their origin to reactive oxygen species (ROS) intermediates. Rr and Dr. bacteria of arid regions have the potential to regulate imbalance radicals and evade a higher dose of radiation and oxidation than bacterial species of non-arid regions. Photochemical-activated ROS in Rr bacteria is run through photo-induction of electron transfer. A hypothetical model of the biogeochemical cycle based on solar radiation and desiccation. These selective stresses generate oxidative radicals for a short span with strong reactivity and toxic effects. Desert-inhibiting Rr bacteria efficiently evade ROS toxicity with an evolved antioxidant system and other defensive pathways. The imbalanced radicals in physiological disorders, cancer, and lung diseases could be neutralized by a self-sustaining evolved Rr bacteria antioxidant system. The direct link of evolved antioxidant system with intermediate ROS and indirect influence of radiation and desiccation provide useful insight into richness, ecological diversity, and origin of Rr bacteria capabilities. The distinguishing features of Rr bacteria in deserts present a fertile research area with promising applications in the pharmaceutical industry, genetic engineering, biological therapy, biological transformation, bioremediation, industrial biotechnology, and astrobiology.

## 1 Introduction

Radiation-resistant (Rr) and desiccation-resistant (Dr) bacteria can survive with exposure to radiation and desiccation cycles in natural habitats. Their resistance capabilities were confirmed during laboratory experimentation (Singh and Gabani, 2011; Paulino-Lima et al., 2013), while the radiosensitive bacterial cells immediately reduced in number with radiation exposure (Sghaier, 2011). Based on the wavelength, electromagnetic radiation comprises ionizing radiation (IR) and non-IR. The wavelength of IR falls below 100 nm and includes X-rays and gamma rays and is extremely hazardous because it produces ions in the cell. Other IR includes alpha and beta particles with undetectable wavelengths and high penetrating ability (Griffiths, 2020; Chaudhary and Kumar, 2023). Non-IR does not result in the formation of ions but includes ultraviolet radiation (UVR), whose wavelengths fall between 100 and 400 nm (Mba et al., 2012; Leszczynski, 2014). These Rr bacteria of desiccated soil evolved in response to photo-irradiation and can survive with exposure to radiation environments. Due to selective stresses that prevail in deserts, they are regarded as a unique environment for studying ecological diversity and function potentiality of Rr bacteria. In a desiccated environment, the photochemical production of ROS interlinks defensive pathways against radiation and desiccation, and bacterial communities can survive multiple stress conditions.

Desiccation is a typical feature of the desert’s environment and an important factor that helps microbial cells evolve with resistant features. Microbial communities of deserts have resistant deoxyribonucleic acid (DNA), efficient proteomic systems, richness in metabolites, enzymes, and dissimilar pigmentation (Castillo and Smith, 2017; Orellana et al., 2020; Kanekar and Kanekar, 2022). Desiccated regions such as the Taklamakan Desert, Sonoran Desert, Sahara Desert, and Atacama Desert are extensively studied for their rich bacterial diversity with extreme resistance to radiation, desiccation, and oxidation (Sajjad et al., 2017; Belov et al., 2018; Guesmi et al., 2021; Liu et al., 2022). Radiation and desiccation are the main ecological factors affecting the microbial diversity of the desert ecosystem. However, researchers endeavor to reveal the actual factor affecting the evolved abilities of desert-borne bacteria. Scientists contemplate that the radiation tolerance found in bacterial species of desiccated regions is unusual. This is because the intensity of radiation survived by bacteria inhabiting deserts does not even reach the surface of the Earth. In particular, *D. radiodurans* exhibits an ability to resist radiation that is four times greater in magnitude, with no loss in cell numbers, than the highest recorded radiation on earth (Battista, 1997). Rr bacteria of the desert environment, especially *D. radiodurans*, resist 15,000 Gy of gamma, which is explained by its ability to scavenge ROS (Daly, 2006). ROS plays an intermediate role in interlinking resistance mechanisms against radiation and desiccation.

The evolved abilities of Rr bacteria have wide applications in applied sciences. Rr bacteria utilize the imbalanced charge radicals for their energy generation and consumption. As the redox cycle of Rr bacteria evolved and operated with the intensity of radiation, it might be with desiccation. These bacteria managed intracellular induction of electrons by their counter-response to these environmental stresses. The by-products of radiations and desiccation of bacterial cells in the form of charge radicals and their counter-response help in the evolved abilities (Daly, 2006). Rr bacteria also produce distinct pigmentation with absorbance and reflection of incoming radiation. These pigments are important for scavenging radicals produced by radiation and desiccation. Rr bacteria have an efficient antioxidant system, highlighting their importance in chemotherapy and curing lung diseases, the main obstacle of which is overcoming imbalanced radicals. Rr bacteria efficiently balance elevated radicals that are induced by radiation, desiccation, or any other stress factor (Latifi et al., 2009; Yoboue et al., 2014; Fagliarone et al., 2017; Han et al., 2020). Rr bacteria resist osmotic and acid stress temperature variation and have a pool of enzymes, making them a suitable candidate in fermentation biotechnology. In addition, Rr bacteria exhibit tolerance to heavy metals, playing a crucial role in the bioremediation of pollutants and the biodegradation of radioactive and radionucleotide waste (Luo et al., 2014; Beblo-Vranesevic et al., 2017; Chen et al., 2021; Nayak et al., 2021; Kumar et al., 2022). Furthermore, Rr bacteria produce various extremolytes, and their potential sunscreen ability is already commercialized and effectively utilized by skincare industries (Mendes-Silva et al., 2020).

Therefore, it is evident that the interaction of Rr and Dr. bacteria with various stresses is interesting to study further. Thus, the current review summarized the most recent knowledge about the origin, abundance, and diversity of Rr bacteria in response to radiation and desiccation. This article also covers the following topics: (i) desert environmental traits, (ii) a common antioxidant system by which Rr bacteria sustain in radiation and desiccated environment, and (iii) the potential application of Rr bacteria in the field of biotechnology, bioremediation, biological therapy, fermentation, energy, and pharmaceutical industry.

## 2 Environmental traits of deserts and their influence

### 2.1 ROS is elicited through radiation and desiccation

**Figure 1:**
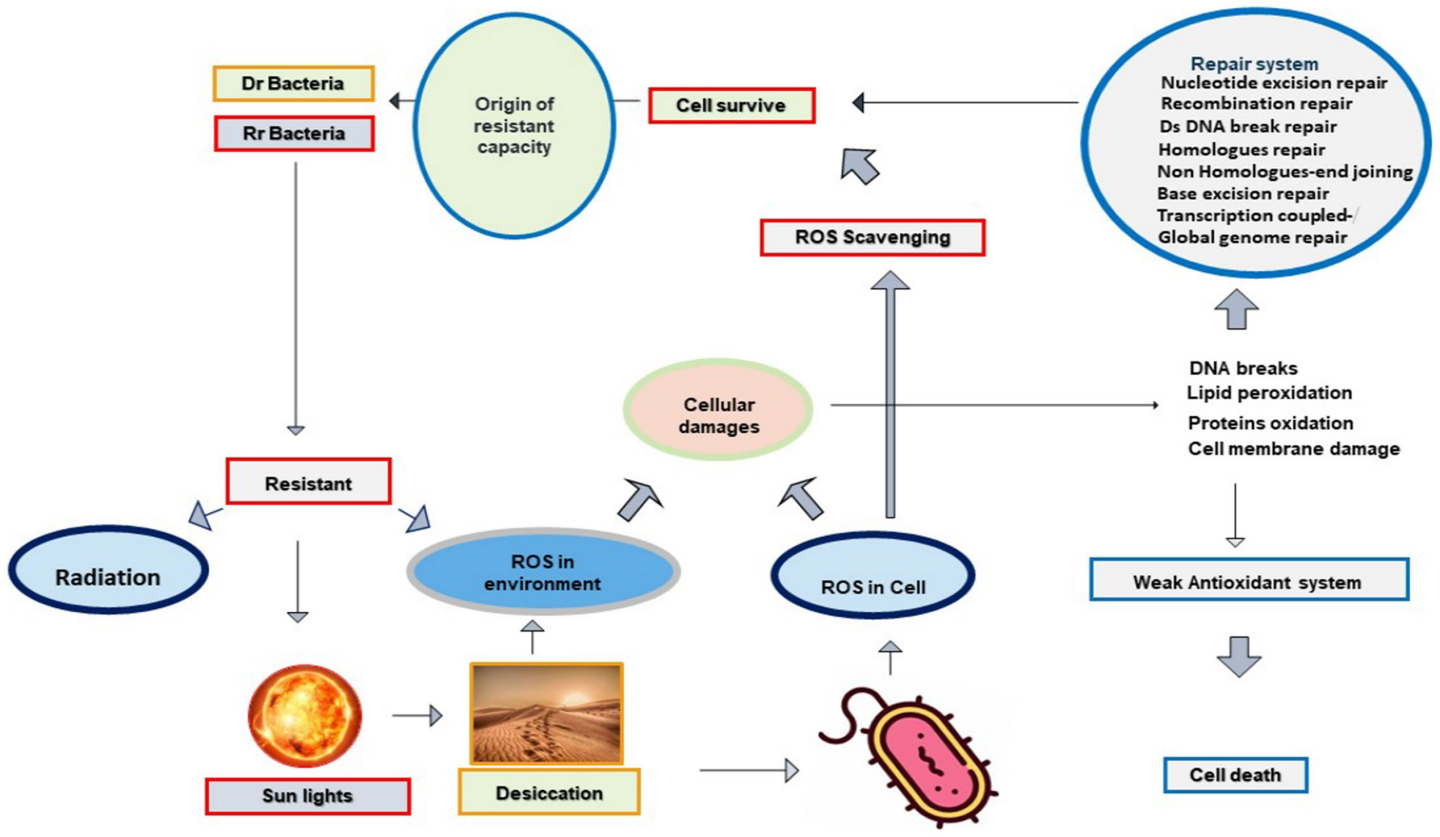
Radiation and desiccation initiate the origin of Rr and Dr. capabilities in deserts through the antioxidant system and repair mechanisms: Radiation and desiccation triggered the production of ROS species in bacterial cells. Due to their strong reactivity, ROS causes DNA damage, protein oxidation, and lipid peroxidation. Bacterial cells having no proper antioxidant system lose their viability and cells marked with evolved antioxidant systems survive with exposure to radiation, desiccation, and oxidation.

Desiccated soil exposed to photoradiation generates ROS species in the desert environment. ROS is also produced inside a cell in response to radiation and desiccation. These selective environmental stresses influence essential components and cellular processes of microbial cells directly or indirectly through ROS derivatives. These ROS are reactive, damage biological molecules with strong affinity, and interfere with cellular processes. ROS interferes with the genome, nucleotides, mitochondrial DNA, proteins, lipids, and the redox cycle of the target cell (Krisko and Radman, 2010; Daly, 2012; Imlay, 2013, 2015; Schieber and Chandel, 2014). Despite their toxicity on cellular contents, a high abundance of microbial communities have evolved ROS scavenging mechanisms with the ability to resist desiccation, radiation, and their derivatives. It is postulated that similar mechanisms work in both cell corrosion and cell protection through ROS intermediates (Figure 1).

### 2.2 Photochemical redox reactions

ROS are produced in the cell and are also found in the surrounding desert environment. These ROS drive the abundance, diversity, and origin of the Rr and Dr. bacteria. ROS are generated by solar radiation and desiccation. Normally, photodesiccated soils accumulate peroxides and superoxide at higher levels than non-desert soils. A study highlighted evidence of superoxide (O_2_^¯^) and hydroxyl (-OH) production in desert soil (Georgiou et al., 2015). Nitrogen oxide (NOx) is released from the desiccated soil of the Mojave Desert at rates comparable to wet soil if the soil is irradiated by solar radiation. This shows the importance of radiation and desiccated soil in regulating ROS and reactive nitrogen species (RNS) (Austin and Vivanco, 2006; McCalley and Sparks, 2009). However, due to the short lifespan of reactive species and the lack of suitable techniques for their detection in the soil, these photochemical oxidative reactions and their associated mechanisms are not fully understood (Ito et al., 1985; Schneider et al., 2014; Halliwell and Gutteridge, 2015).

## 3 Hypothetical models of Rr bacteria origin in deserts

### 3.1 A naturally occurring desiccation

The most accepted and main driving force for the origin of Rr bacteria is desiccation. Rr bacteria are naturally resistant to desiccation. It is known that desiccation and radiation share a common target in a bacterial cell by causing the disintegration of the DNA molecules (Singh, 2018), while in response, Dr. and Rr bacteria also effectively recover the lesions of DNA caused by either desiccation or radiation. The similar target of radiation and desiccation in the cell and the similar mechanism of the cell to evade damaging effects in both conditions give a clear understanding that the desiccated environment of deserts is one of the important aspects of the rich diversity of Rr bacteria (Rainey et al., 2005). The hypothesis that Rr Bacterial community is a consequence of desiccation was previously studied. It was shown that resistance to a high radiation dose and desiccation is regulated through ROS scavenging system and efficient DNA repair mechanisms. It was shown that such mechanisms compensate for desiccation and also evolved to resist radiation (Makarova et al., 2001; Cox and Battista, 2005; Yu et al., 2015). To correlate Rr bacteria with desiccation, a phylogenetically diversified Rr bacteria belonging to *Bacteroidetes*, *Proteobacteria*, *Deinococcus-Thermus*, Firmicutes, and *Actinobacteria* were studied from the Taklimakan Desert (Yu et al., 2015). Interestingly, all Rr bacteria have the potentiality of carotenoid-like molecules, an efficient scavenger of ROS and reactive nitrogen species (RNS) (Asker et al., 2012). These ecologically distributed Rr bacteria and their associated ROS scavengers possess higher activities against desiccation and oxidation. Research on the desiccated environment of Taklimakan supports the hypothesis that the resistant phenotype is a consequence of the evolution of the ROS scavenging system, which defends cells against oxidative damage caused by desiccation (Yu et al., 2015).

### 3.2 A naturally occurring radiation

The Rr bacteria at desiccated habitat balance radicals generated by radiation at the atomic level. This mechanism is based on the availability of free electrons with ROS species and their immediate balancing. These ROS species and their unstable revolving electron are hit by incoming radiation. These electrons are excited after they gain energy from the incoming photon; these electrons circulate freely in the cytoplasm and damage cellular structures, commonly DNA, lipids, and proteins. Due to available free electrons, ROS species are very reactive and damage biological and cellular structures (Atri et al., 2022). To balance charge particles caused by radiation, these ROS species have to lose or gain more charge ions either to build a new molecule or convert the toxic to a less toxic form. In simple words, this redox cycle is imbalanced by photon–electron interaction through ROS intermediates and balanced again by an antioxidant system of Rr bacteria. Due to this evolved feature, Rr bacteria are more resistant than radiosensitive cells (Tiquia-Arashiro and Rodrigues, 2016).

Photosynthetic cells also have a photo-induced electron cycle at the atomic level. The electrons derive excitation as soon as the incoming photons collide with the electrons in the antenna complex of the photosystem. These excited electrons are consumed in photophosphorylation (Kramer and Evans, 2011; Maróti et al., 2020). Photon energy from electromagnetic radiation transforms visible light into electrical energy and chemical energy (Chandra et al., 2018). Bacteria inhibiting in desiccated soil are naturally pigmented in response to radiation. Biologists assume that the Rr bacterial community of deserts drives this useful phenomenon at the cellular level in response to radiation. The radiation (photon–electrons) interaction or ionic interaction mediates the flow of charge radicals in the cell. Thus, it is concluded that radiation is a source of energy for physiological function and also the transformation of energy in the cell from one form to another (Shukla and Subba Rao, 2017), while a considerable amount of radiation converts to heat, as an answer is why the Rr bacterial community evades from a dose higher than the natural radiation.

### 3.3 Temperature, salinity, and Rr bacteria

The extreme Rr bacteria and rich diversity of desert-borne bacteria also have temperature dependency. At freezing temperature, the ROS produced due to radiation is restricted to diffuse, whereas, at room temperature, the ROS produced freely moves and disintegrates DNA molecules higher than ROS at freezing conditions. The gamma radiation resistance of *D. radiodurans* and isolated strains from the extreme cold desert of Antarctica Dry Valley was compared under varying temperatures. *D. radiodurans* withstand a high dose of gamma radiation at −79^°^C when frozen on dry ice compared to room temperature (Dartnell et al., 2010). The IR resistance of microbes is also studied with correlation to salinity. There is no correlation between high Rr and Dr. bacteria with salt concentration (Shukla et al., 2007).

## 4 Geographical distribution of Rr bacteria

Rr bacterial communities are ecologically distributed in hot and cold deserts (Chanal et al., 2006; Shukla et al., 2007; Azua-Bustos et al., 2012; Musilova et al., 2015; Shirsalimian et al., 2018; Guesmi et al., 2022) *Deinococcus thermus* strains LD4 and LD5 were isolated from the Lut Desert of Iran (the hottest place on earth) and are resistant to a dose of >15 KGy of gamma radiation and > 600 j/m^2^ of UV-C (Mohseni et al., 2014). Their extreme radiation resistance is explained by desiccation and temperature fluctuation during the day and night. Similarly, *Hymenobacter xinjiangensis* X2-1g^T^ has been reported from the Xinjiang Desert, China, which can resist 8 KGy of gamma radiation from a ^60^Co source at a dose rate of 10 Gy min^−1^ (Zhang Q. et al., 2007). *Desertibacter roseus* strain 2622^T^ has been reported from the Xinjiang Taklamakan Desert of China, which can survive at 10 KGy of gamma radiation at a dose rate of 300 Gy min −1 at room temperature (Liu et al., 2011). These species are ecologically dispersed, and the genus *Deinococcus* is widely found in the Deserts. For instance, 60 strains of this genus were isolated from the irradiated arid soil of the Sonoran Desert, including 9 strains identified for the first time (Rainey et al., 2005). In total, 14 Rr strains, such as species of *Bacillus cereus/thuringiensis* of phylum Firmicutes, have been reported from the Atacama Desert. These strains showed 300 j/m^2^ of UV-C tolerance except for *Bacillus* strain S3.300–2, which showed a 10% survival rate even at the higher dose of 318 j/m^2^ (Paulino-Lima et al., 2013). The details of Rr bacteria and their potential to resist radiation are described in Table 1. Due to harsh ecological elements prevailing in deserts and most commonly fluctuations of chemical and physical weathering, Rr strains have evolved to produce antiviral, antibacterial, and antifungal metabolites. As a result of rapid emerging resistance against the preexisting antibiotics, the search for new metabolites obtained from such extreme habitats is crucial to contain human and animal diseases (Gabani and Singh, 2013; Nayak et al., 2021). Rr bacteria are not only resistant to oxidation and radiation but survive with fluctuation of temperature and are categorized into mesophile, thermophile, and hyperthermophile subgroups (Ranawat and Rawat, 2017; Li et al., 2020; Marszalkowski et al., 2021). These bacteria are found in less non-arid habitats than in arid ones, as plenty of photochemical production of ROS in the desert ecosystem facilitates their origin, diversity, and abundance (Rainey et al., 2005). It is therefore concluded that Rr bacteria is found in soil either irradiated or desiccated and eventually grows by following a cycle of oxidative regulation (Schulze-Makuch et al., 2017).

**Table 1:** Rr bacteria and their resistance capabilities: Rr bacteria isolated from various desiccated soil across the world.

Desert bacterial strains	Isolation source	UVC survival rate/dose	UV-B survival rate/dose	Gamma survival rate/KGY dose	References
*Deinococcus deserti* (VCD117)	Tunisia Sahara Desert sand	11%/250 J m^−2^ (LR)		60%/2.5, 15%/5, and 6%/7.5 of KGy dose, respectively (LR).	De Groot et al. (2005)
*Deinococcus Saudiensis* (YIM F235) & (YIM F302T)	Saudi Arabia Medina province Desert of Yanbu’ al Bahr		35.5 and 25.6% at 5 J m^−2^ for YIM F302^T^ and YIM F235, respectively (LR)	68.0 and 21.0% at 2.5 KGy, 44.0 and 14.0% at 5 kGy for strains YIM F302^T^ and YIM F235, respectively (LR)	Hussain et al. (2016)
*Deinococcus taklimakaensis* (X-121^T^)	China, Xinjiang, Taklamakan Desert Soil	2.3%/460 J m^−2^		7% 10 KGy (IR)	Liu et al. (2017)
*Deinococcus gobiensis* (1-0^T^)	China Xinjiang Gobi Desert	Resistant >600 J m^−2^		Resistant >15KGy of KGy (HR)	Yuan et al. (2009)
*Deinococcus radiopugnans* (WMA-LM9)	Pakistan Lakki Marwat Desert		79.47%/3.30 × 10^3^ J m^−2^ (HR)		Sajjad et al. (2018)
*Deinococcus xinjiangensis* (X-82^T^)	China Xinjiang	.		1–0.5%/5 KGY(LR)	Peng et al. (2009)
*Deinococcus peraridilitoris* (KR-196), (KR-198), (KR-200 T)	Chile’s coastal Desert, Arid soil sample			Resistant >10 KGy (IR)	Rainey et al. (2007)
*Desertibacter roseus* strain 2,622 T	China Xinjiang Taklamakan Desert			Resistant to 10 KGy (IR)	Hezbri et al. (2016)
*Geodermatophilus pulveris* (BMG 825^T^)	Tunisia Sahara Desert Limestone dust			10%/9KGy(IR)	Hezbri et al. (2016)
*Hymenobacter xinjiangensis* (X2-1gT)	China Xinjiang Desert			Resistant to 8 kGy (IR)	Zhang Q. et al. (2007)
*Deinococcus deserti* (VCD115^T^)	Morocco Sahara Desert sand sample		73% /250 J m^−2^ (HR)	95%/2.5, 94%/5, and 23%/7.5 of KGy dose, respectively (IR)	De Groot et al. (2005)
*Maritalea* (A2) *Maritalea* (B2)	Iran Lut Desert, Gandom Beryan region			D10 value b/w 2 and 4 KGy (LR)	Shirsalimian et al. (2018)
*Pseudomonas stutzeri* (S4.100–3)	Atacama Desert Sitio 2 Gypsum	Tolerance of 300 Jm^−2^ (IR)			Paulino-Lima et al. (2013)
*Staphylococcus lugdunensis* (WMA-BD4)	Pakistan Bahawalpur Desert		48.27%/2.0 × 10^3^ J m^−2^ (HR)		Sajjad et al. (2018)
*Stenotrophomonas maltophilia* (WMA LM10)	Pakistan Lakki Marwat Desert		46.15%/1.30 × 10^3^ J m^−2^ (HR)		Sajjad et al. (2018)
*Stenotrophomonas* sp. (WMA-LM19)	Pakistan Lakki Marwat Desert		51.69%/1.30 × 10^3^ J m^−2^ (HR)		Sajjad et al. (2018)

Low resistance (LR) refers to 10% survival at 1–5 KGy of gamma radiation and < 300 J m^−2^ UVC and < 1,000 J m^−2^ UV-B. Highly resistant (HR) refers to 10% survival at >10 KGy gamma radiation, >500 J m^−2^ UVC, and > 1,500 J m^−2^ UV-B. Intermediate resistance (IR) refers to 10% survival at 6–10 KGy of gamma radiation and 300–500 J m^−2^ UVC and 1,000–1,500 J m^−2^ UV-B.

## 5 Mechanism for ionizing radiation resistance

### 5.1 Copy number of DNA and nucleoid organization

IR resistance in bacteria has focused on explaining how cells cope with DNA and protein damage and detoxify ROS (Villa et al., 2021; Rai and Dutta, 2024). DNA and protein damage are believed to have the most significant impact on cell survival. Increased DNA copy number has been positively correlated to increased IR resistance in *E. coli* (Krasin and Hutchinson, 1977) and *Saccharomyces cerevisiae* (Mortimer, 1958). Increased numbers of DNA copies provide enough genetic information that DNA repair systems can use to correct the damages caused by IR. Recombination, a process that bacteria frequently use to repair DNA double-strand breaks (DSBs), requires more than one copy of the DNA being repaired (Cox and Battista, 2005). The nucleoid of IR-resistant bacteria, including *Rubrobacter radiotolerans* and *Deinococcus* spp., appears to be more condensed than the genome of IR-sensitive species such as *Thermus aquaticus* and *E. coli* (Zimmerman and Battista, 2005). The condensation prevents DNA fragment diffusion and limits the post-irradiation activity of intracellular DNases, thus facilitating cell survival.

### 5.2 Enzymatic and non-enzymatic processes and proteins

The oxidative stress caused by IR is prevented through enzymatic processes. The activity of the following proteins protecting against oxidative stress (peroxidases, catalases, and superoxide dismutases) relates to ROS accessibility and may be a contributive factor for IR resistance (Wang and Schellhorn, 1995; Tian et al., 2004). As for non-enzymatic processes, antioxidant molecules, including carotenoid pigments and intracellular salts, are involved in protection against oxidative stress. Novel proteins in *D. radiodurans* have been linked to IR resistance. Five novel protein genes are highly expressed and encoded proteins of unknown function in *D. radiodurans* following exposure to IR, such as DdrA, DdrB, DdrC, DdrD, and PprA. These proteins mediate Rec A-independent processes related to IR (Harris et al., 2004; Tanaka et al., 2004).

### 5.3 Radiation resistance mechanism against UV radiation

DNA damage caused by UV radiation is dependent on wavelength. UV-A (320 to 400 nm) induced only indirect damage to DNA, proteins, and lipids through ROS species intermediates. UV-B radiation (280 to 320 nm) and UV-C (100 to 280 nm) cause direct and indirect damage. The most common products formed by UV-B irradiation are cyclobutane pyrimidine dimers (CPD) (Mitchell and Karentz, 1993). Rr bacteria have numerous repair mechanisms in response to damage caused by UVR. These mechanisms are divided into photoreactivation and dark repair (DR). There are a total of three different dark repair mechanisms (i) nucleotide excision repair, (ii) error-prone repair, and (iii) postreplication recombinational repair (Fernández Zenoff et al., 2006).

## 6 Potentiality and diversity in applications of Rr bacteria

**Figure 2:**
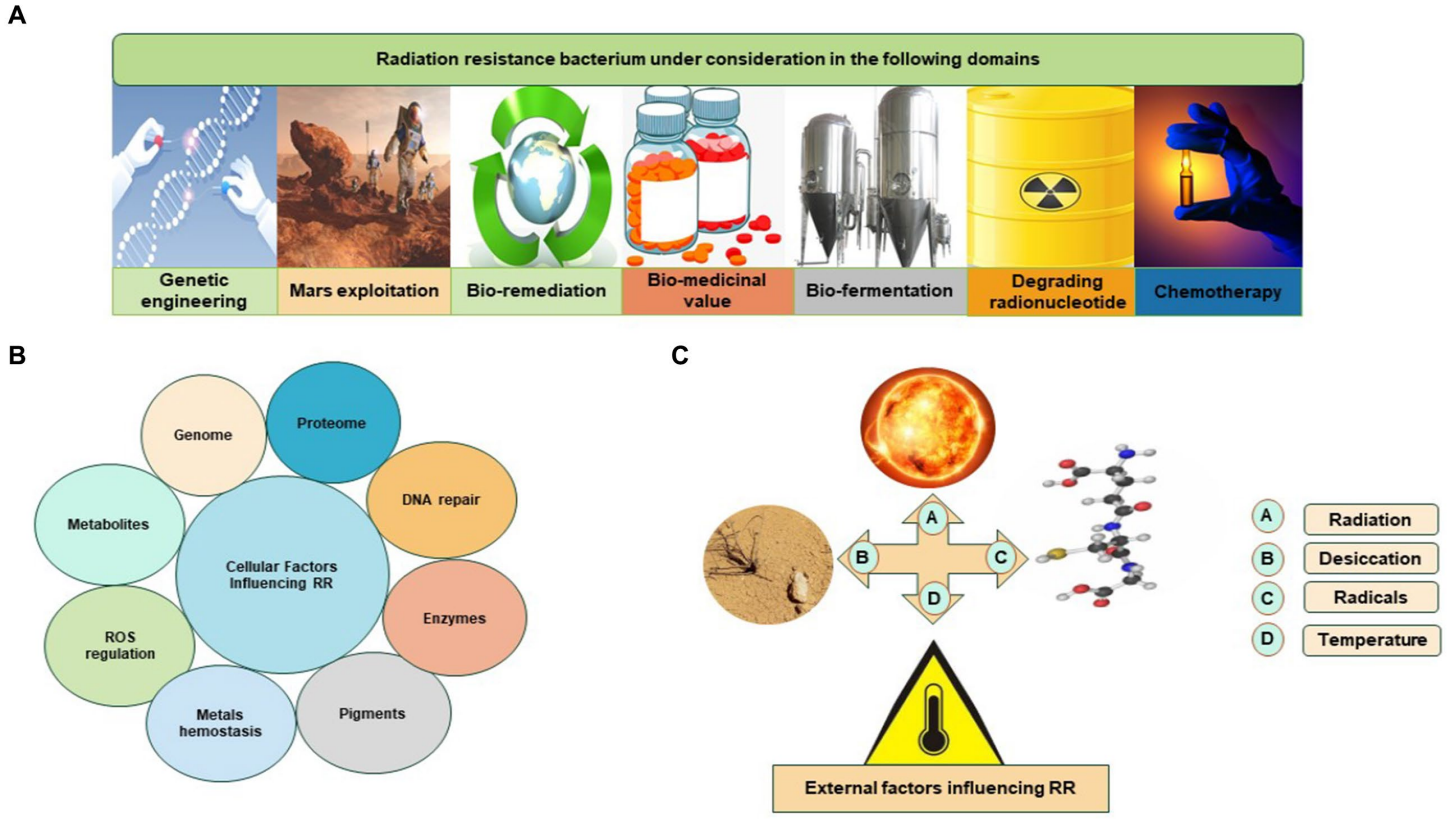
Rr bacteria is under different considerations: **(A)** Rr bacterium in various sectors; **(B)** cellular factors influencing Rr bacteria; **(C)** external factors influencing Rr bacteria.

The Rr bacteria are under consideration in different domains (Figure 2). The extraordinary capabilities of Rr bacteria are defined by known features such as radiation resistance, regulation of ROS, antioxidation, DNA repair mechanism, efficient proteomic system, enzymes, and metabolites such as carotenoid pigments. These features of Rr bacteria are very effective in natural conditions. These natural traits of Rr bacteria can be manipulated and cloned by modern biotechnological approaches. Rr bacteria could be utilized for their importance in the pharmaceutical industry, lung disorders, chemotherapy, bioremediation, fermentation, radioactive waste management, and biodegradation of biomass to yield valued-added compounds (Kumar et al., 2010; Guan et al., 2017; Nayak et al., 2021; He et al., 2022).

### 6.1 Antioxidant system of Rr bacteria

The antioxidant system of Rr bacteria efficiently protects cells from internal and external stresses. The antioxidant system of Rr bacteria has both enzymatic and non-enzymatic pathways. The homeostasis of metal ions (Peana et al., 2018) further strengthens the antioxidant system of bacteria. Metal ions such as Cu^2+^, Mn^2+^, and Zn^2+^ are important to balance free radicals in bacterial cells exposed to UV-B radiation (Santos et al., 2013). Rr bacteria regulate intracellular Cu^2+^, Mn^2+^, and Zn^2+^. These ions reduce peroxide stress by blocking the Fenton and Haber–Weiss reactions (Bagwell et al., 2008; Daly et al., 2010). The cellular metabolism of Rr bacteria is least affected by environmental stresses with evolved antioxidant systems (McLean and McLean, 2010; Etemadifar et al., 2016). Rr bacteria with efficient antioxidant systems are less, vulnerable to protein oxidation, lipid peroxidation, and nucleic acid breaks with radiation, desiccation, and oxidation than radiosensitive bacteria (Sajjad et al., 2018).

### 6.2 Non-enzymatic antioxidants and their applications

**Figure 3:**
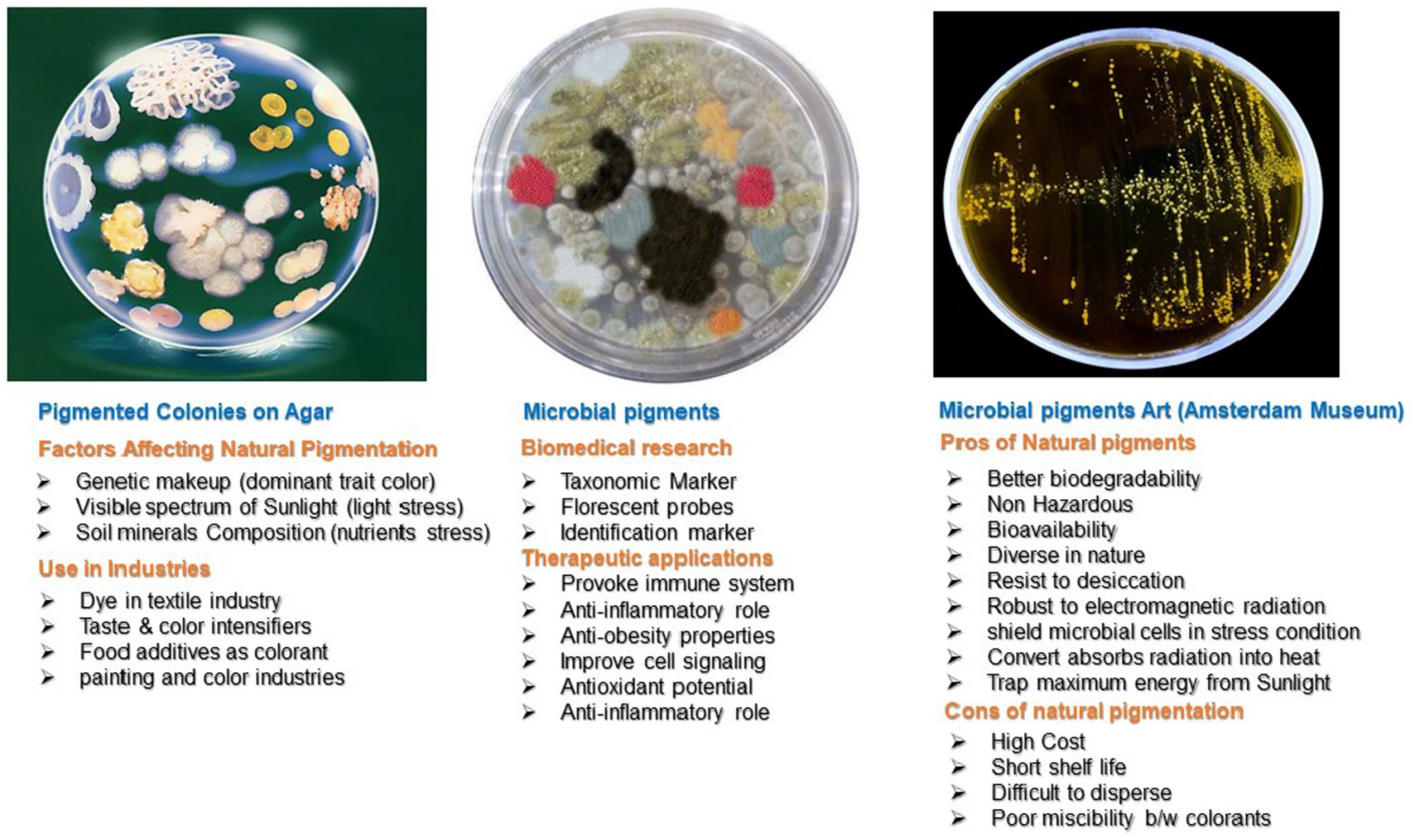
Pros and cons of natural bacterial pigments and their applications.

Carotenoids are non-enzymatic antioxidants (Krinsky and Johnson, 2005; Bing Tian et al., 2007). Carotenoids are natural pigments, tetra-terpenoids including C40 hydrocarbon backbones (Carotenes) and their oxygenated derivatives xanthophylls. Carotenoids have a major role in radioresistance due to their efficient ROS scavenging ability (Yang, 2021). These ROS include hydrogen peroxide (H_2_O_2_) singlet molecular oxygen (^1^O_2_), -OH, O_2_^¯^, and RNS, such as 2,2-diphenyl-1-picrylhydrazyl (DPPH^•^) and nitric oxide (NO) (Tian et al., 2009). Microorganisms and plants manufacture carotenoids and have wide applications (Figure 3). *D. radiodurans* and other bacterial species synthesize different types of carotenoids (Krinsky and Johnson, 2005; Asker et al., 2012). The 13 genes of *D. radiodurans* are involved in the biosynthesis of red color carotenoids (Saito, 2011). However, bacteria produce carotenoids of different colors. The pink, yellow, and red color appearance of Rr isolates from the Taklamakan Desert is due to the production of carotenoid-like molecules (Yu et al., 2015). A unique carotenoid deinoxanthin efficiently scavenges O_2_^¯^and H_2_O_2_. Deionxanthin prevents protein oxidation at lower concentrations and has potent scavenging ability than lycopene and alpha-carotene (Zhang L. et al., 2007). These pigments and metabolites are responsible for the photoprotection of cells against UV light in deserts (Yu et al., 2015). Carotenoid pigments are significant to human health. They are efficient quenchers of reactive O_2_ and are linked to several diseases in humans, such as life-threatening cancer and other chronic disorders. The epidemiological data on carotenoid supplementation showed that ROS-mediated disorders are largely controlled by their consumption in a normal diet. Carotenoids regulate the balance of free radicals in a cell and are potential supplements for aging control, cancer prevention, and other beneficial aspects of human health (Burda, 2014; Rivera-Madrid et al., 2020).

### 6.3 Enzymatic antioxidants and curative measures of the lungs

Rr bacteria produce various enzymes, such as catalase (CAT) and superoxide dismutase (SOD), and some species also possess oxidases. These enzymes scavenge free radicals and ions. ROS detoxification is vital for the survival of the irradiated cell. Therefore, bacterial cells evolved to yield various enzymes to maintain the balance of ROS production and their removal. These enzymes are the first line of defense to protect microbial cells from radiation. *D. radiodurans* enzymatic antioxidants CAT and SOD are involved in the chemical conversion of ROS to less toxic substances (Markillie et al., 1999; Makarova et al., 2007).


(1) $$2O^{2-}+2H^{+}\to H_{2}O_{2}+O_{2}\text{.}$$

(2) $$2H_{2}O_{2}\to 2H_{2}O+O_{2}\text{.}$$

Rr bacteria evolved with such efficient enzymatic pathways could, fortunately, serve as a supplement in the injured lung tissue. These enzymes can potentially detoxify harmful radicals caused by smoking or any other inhaled chemical inducer. A balance of oxidant and the antioxidant system is important in the cellular hemostasis of normal lung tissue, but physiological disturbance occurs when the antioxidant system fails to neutralize a high amount of ROS. These lung complications include chronic obstructive pulmonary disease (COPD) and asthma (Leibel and Post, 2016). Cigarette smoking causes nearly 90% of all lung cancer deaths and 80% of COPD, such as emphysema and chronic bronchitis. An innovative approach involving these enzymes is crucial to overcome lung disorders caused and worsened by a high amount of ROS. A competent Rr bacteria evolved with an enzymatic antioxidant system is important to control ROS toxicity in lung disorders, which might overcome radicals and prevent their entry into blood through air sacs and other body tissue. ROS induces oxidative damage to DNA, carbohydrates, lipids, and proteins, initiating an array of downstream processes promoting the progression of COPD in affected lungs. The genetic ability of Rr bacteria against oxidative damage could be engineered as probiotics supplementing to treat smoking lung disorders.

### 6.4 Pharmaceutical products

The extremolytes of Rr bacteria include mycosporine-like amino acids (MAAs), systonemin, bacterioruberin, ectoine, sphaerophorin, melanin, pannarin, and polyhydroxyalkanoates (PHA). These extremolytes reduce photodamage associated with UV radiation. The enhanced potential sunscreen ability of MAA is already commercialized and effectively utilized for skin care industries (De la Coba et al., 2009; Slaninova et al., 2018; He et al., 2022). Extremolytes such as asterina, palythine, palythene, and palythinol indicate anticancer and skin care products (Llewellyn and Airs, 2010). Scytonemin may act as an anti-inflammatory and antiproliferative drug to offer a novel pharmacophore for protein kinase inhibitors. Scytonemin has been shown as a competitive ATP inhibitor of Polo-like kinases (PLKs) (Murugan et al., 2011). PLKs have been the cancer target for many years because PLKs control many oncogenes. Previous studies have shown that scytonemin has a major role in hyperproliferative disorder because of its ability to inhibit PLK1 (Stevenson et al., 2002). Another research showed PLK1 inhibition mediated by scytonemin-induced apoptosis of cancer cell types such as osteosarcoma (Strebhardt and Ullrich, 2006; de Cárcer et al., 2007; Duan et al., 2010), suggesting that scytonemin is a promising anticancer agent. Similarly, ectoine also has therapeutic potential; for example, the human kidney keratinocytes irradiated with UV-A were effectively treated with ectoine. Ectoine has also been studied for skincare applications against desiccation, water loss, and UV damage. Due to water loss, ectoine is a prophylactic agent for dry skin (Buenger and Driller, 2004). Ectoine was also observed to prevent the damage associated with bacterial lipopolysaccharide (Buommino et al., 2005). *Rubrobacter radiotolerans* produce another compound, bacterioruberin, which prevents cancer and repairs the DNA breaks caused by IR in human cells.

### 6.5 Rr bacteria impact on cancer control

Oxidative stress is linked with cancer, a physiological disorder. Rr bacteria scavenge imbalanced radicals and potentially prevent protein oxidation and DNA damage. *D. radiodurans* could be a model bacteria for cancer control because of their resistance to extreme radiation levels, desiccation, and other oxidative stress conditions (Slade and Radman, 2011; Krisko and Radman, 2013; Qi et al., 2020). High levels of ROS promote disease pathological conditions. These ROS stimulate the apoptosis of cancer cells and are efficiently utilized as a chemotherapeutic tool (Li et al., 2011; Redza-Dutordoir and Averill-Bates, 2016). Recently, a study highlighted the role of crude secondary metabolite extract (CSME) of *D. radiodurans* in triple-negative human breast carcinoma MDA-MB-231 cells. CSME-induced ROS production encourages nuclear membrane alterations with apoptotic destruction of MDA-MB-231 cells. *D. radiodurans* CSME upregulates apoptotic marker expression in breast cancer chemotherapy (Maqbool et al., 2020). Further study is required to explore the role of CSME as a bioactive compound for cancer control on a molecular level.

### 6.6 Fermentation of organic waste

The Rr microbes that are utilized for the production of different useful compounds, for example, use the recombinant *D. radiodurans* for the fermentation of organic waste, particularly lignocellulosic biomass (Jiang et al., 2017) to produce value-added compounds. Using its extremophilic properties and resistance to many stresses, like heavy metal resistance, pollutants resistance, osmotic and acid stress, temperature, and the ability to resist DNA-damaging agents of this host will help in survival in fermentation along with a high expression of the desired pathway and high production. The engineering of this host for the pretreatment of lignocellulosic biomass can be coupled with physicochemical pre-treatments such as radiation and acid–alkali under different conditions (temperature, suspension pH, and nutrient availability).

### 6.7 Efficient expression system of Rr bacteria

Rr bacteria could also be utilized as an expression system to clone desired products. The deep genome study of *D. radiodurans* has provided insight into the genetic makeup for tolerating multiple stress conditions. The presence of different pathways and some unique genomics ability to uptake genetic material from outside has made this bacterium suitable for use in a different application through genetic engineering (Makarova et al., 2001). The introduction of novel bioremediation capabilities into *D. radiodurans* has been successfully achieved. Chromosomal integration and vector-based expression of foreign genes in trans have proved effective (Misra et al., 2012). Using such multiresistant microbes will help to overcome the limitations of the enzymatic processes, such as the high costs of the pure enzyme, the fragile nature of the enzyme, very narrow reaction conditions, the low half-life of the enzyme, and the need for exogenous cofactors. Instead of using regulators and genes from *D. radiodurans* (Pan et al., 2009), introducing the whole pathway in *D. radiodurans* can help increase the desired product’s expression in extreme conditions. Due to the remarkable DNA repair mechanism, engineering this host will help to design a process where other microbes are unable to survive due to the presence of DNA-damaging agents.

### 6.8 Energy conversion by Rr bacteria

**Figure 4:**
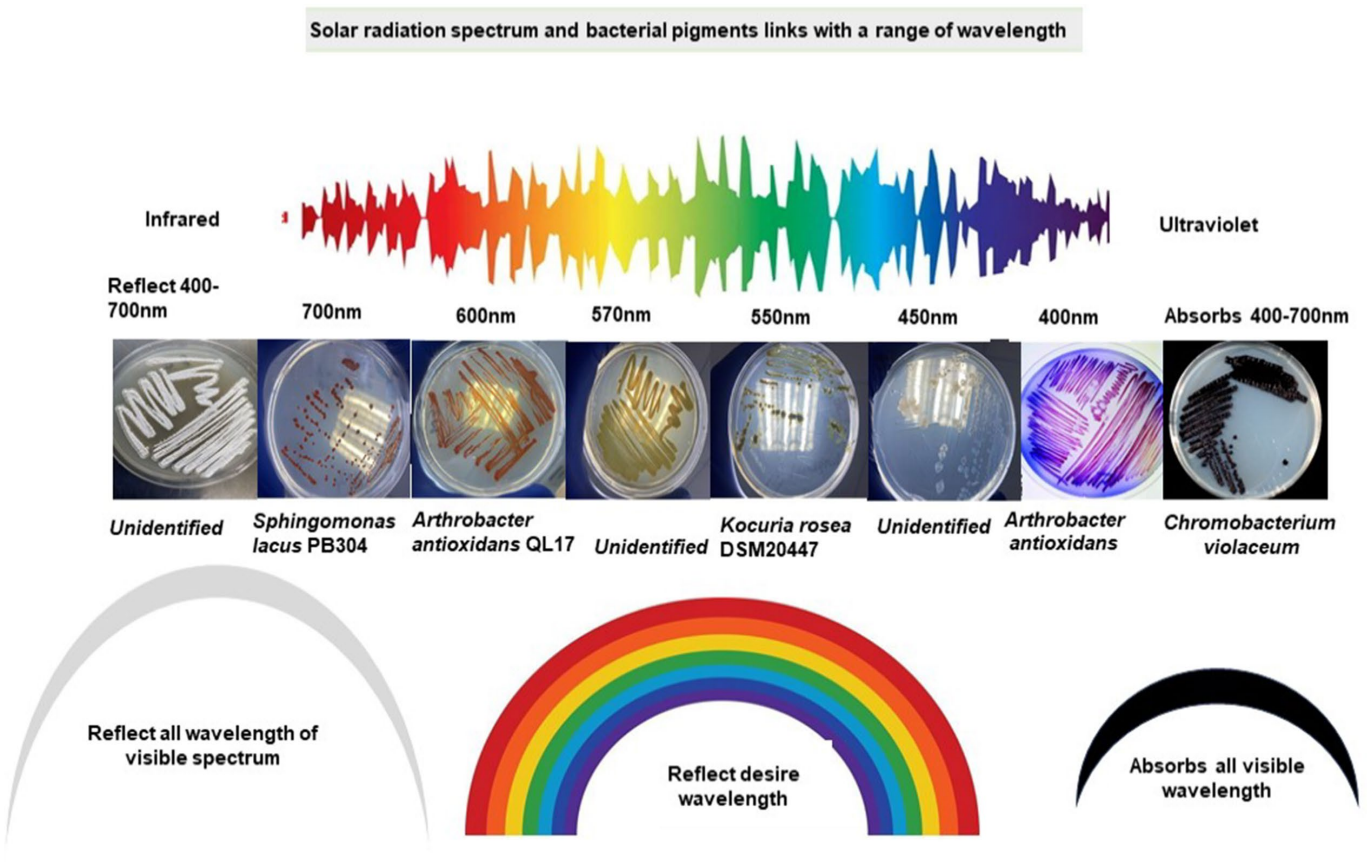
Visible spectrum of solar radiation and characteristics of bacteria pigments with a range of wavelengths.

Rr bacteria have distinctive morphological pigmentation. Rr bacteria abundantly display red, yellow, pink, orange, or simultaneous contrast such as red-orange or yellow-green characteristics colors. These features and colors are inclined from one trait to another (Kreusch and Duarte, 2021; Liu et al., 2022). Most of the reflected colors match the wavelength specified for the respective range of the visible light spectrum. This raises an interesting scientific query to investigate whether color rich diversity of the arid habitats evolved through the influence of different wavelengths of electromagnetic radiation (Figure 4). The characteristic color of bacteria is due to the reflected spectrum of radiation, which cannot be absorbed by a cell. Meanwhile, the absorbed radiation is a driving force for cellular processes, energy generation, and consumption from raised ROS species. A considerable amount of radiation also dissipates as thermal energy in the form of heat (Castillo et al., 2015; Makhneva et al., 2020). Bacteriochlorophylls (B) Chls and carotenoids are bound non-covalently to specific apoproteins, as the main light-harvesting and energy-transforming pigments of photosynthetic organisms. B Chls are vital components of the photochemical reaction centers and account for most of the antenna pigments in anoxygenic photosynthetic bacteria. Due to electromagnetic exposure, the excitation reaching the photochemical reaction centers of B Chls at the lowest singlet excited state causes initial charge separation. Thus, the conversion of light energy into electrochemical energy is initiated and ultimately provides a driving force for all essential processes in photosynthetic and heterotrophic organisms (Renger, 2007).

### 6.9 Radioactive waste management through Rr bacteria

**Figure 5:**
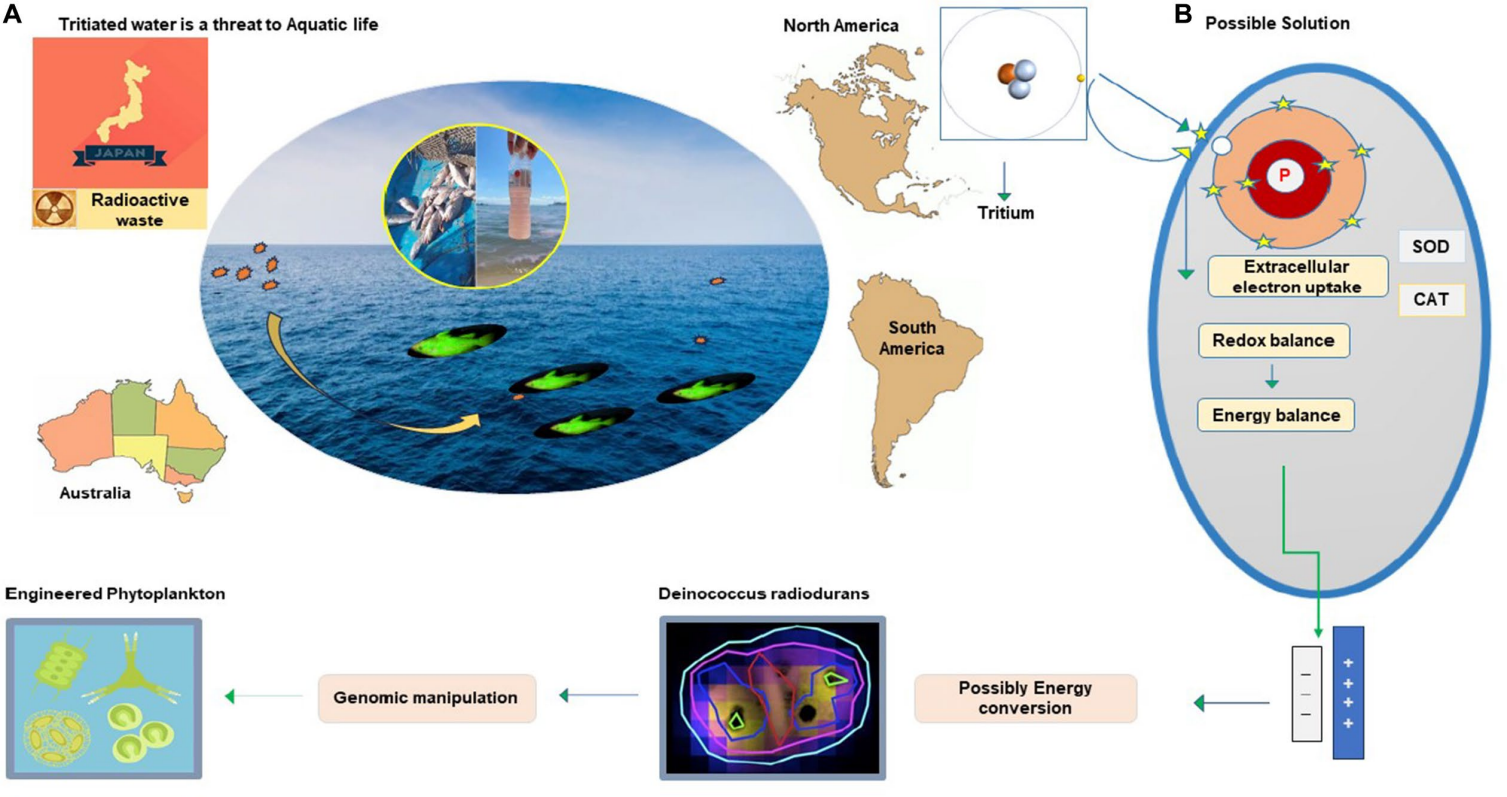
Radioactive waste degradation at the contaminated site by utilizing the antioxidant potential of Rr bacteria.

The world is concerned about discharging radioactive waste from anthropogenic sources before it is converted to less hazardous or fully non-hazardous form. Recently, alarming news about Fukushima tritium discharge in the marine ecosystem was circulated. This raised responsiveness about the risk to aquatic life and the food chain it disturbs. Reconsidering and global management of both natural and anthropogenic reservoirs of tritium discharge in the ocean and its impact on aquatic life is necessary. The insight of earlier studies has revealed the effects of the tritium on natural biota and the potential risk to human health it poses through the food chain (Galeriu et al., 2008; Melintescu and Galeriu, 2011; Zhao et al., 2021). Tritiated water has 99% of tritium, rapidly reaching the ocean through precipitation, river runoff, and evaporation (Liger et al., 2018; Oms et al., 2019). Three main reactors are located on the shore of Canada’s Great Lakes with a maximum level of tritium (8.4 Bq L − 1) greater than twofold as offshore waters (3.5 Bq L − 1). Non-nuclearized coastal rivers in France have a tritium concentration of 3 to 4 Bq L − 1, but in Rhone River, this concentration varied from 2.50 to 12.85 Bq L − 1 with a mean of 6.31 Bq L − 1, and this is because of high nuclear reactors (Jean-Baptiste et al., 2018). The prevalence of tritium in oceans and other contaminated sites is important to conserve aquatic biota and to reduce its potential risk to human health. This would not been possible without innovative ways. Tritium releases negative charge beta particles from radioactive decay and causes cellular damage if inhaled or ingested (Roch-Lefèvre et al., 2018). Humans acquire a considerable amount of organically bound tritium from aquatic biota, mainly through fishes that rely on zooplankton and phytoplankton for their food cycle. The evolved features of bacterial cells that receive and transform harmful charge radicals into a thermal spectrum are fascinating. This could be utilized to balance charge radicals produced through radioactive decay by introducing its consortium into radioactive sites. Rr bacteria could transform radioactive contents into a non-hazardous form or possibly energy exchange (Figure 5). Another approach is *E. coli,* and *Saccharomyces cerevisiae* are genetic engineering platforms of biotechnological applications. However, due to the specific ability to grow and express novel engineered functions in recent years, research has begun using *D. radiodurans* in biotechnologies (Basu, 2022) and bioremediation (Manobala et al., 2019). Toxic waste management is achieved by successful gene transfer, and expression is reported in *D. radiodurans* for bioremediation of nuclear radioactive and heavy metal-polluted environments (Shukla et al., 2017; Khan et al., 2021). Using *D. radiodurans* regulators in yeast and bacteria enhances its activity, and production is successful in fermentation. These IR-resistant bacteria have achieved their target for radioactive waste degradation and could be utilized for radioactive-contaminated sites (Pan et al., 2009; Ma et al., 2011).

## 7 Gaps and future recommendations

ROS regulation and common defensive pathways in Rr bacteria help to understand the origin of resistance against radiation, desiccation, and oxidation. Rr- and Dr-resistant bacteria found in deserts are important for understanding ROS-associated toxicities in human pathological and physiological conditions. The features of the antioxidant system found in the Rr bacterium can be inserted in human disorders, which are known by a disturbance in the regulation of the redox cycle. Rr bacterium regulates a high level of ROS due to radiation exposure. The sensitivity of human cancer cells to radiation underscores the knowledge of the fact that if bacteria can resist radiation, could human cells also evade doses capable of causing damage? Due to its radio-tolerant nature, the Rr bacterium also opens opportunities for degrading radioactive waste. Rr bacterium is a possible solution to generate useful energy in response to incoming photon flux and a platform for producing novel antimicrobial agents and useful metabolites of commercial significance.

### 7.1 Strength of the review

This review article has mainly emphasized the origin, applications, and geographical distribution of Rr bacteria in extreme desert environments. Their by-products, such as enzymes and non-enzymatic antioxidants, were linked to disease pathological conditions. Desert environmental traits such as the photochemical production of ROS in desiccated soil and their impact on radiation resistance have been highlighted. The review article also summarized Rr bacteria from different geographical locations, and data were collected on their survival potential in varying intensities of electromagnetic radiation. Furthermore, the importance of Rr bacteria was evaluated in several sectors, of which microbial biotechnology, bioremediations, fermentation, the pharmaceutical industry, and biological therapy are mainly described.

### 7.2 Limitation of the review

Extensive research is further required to evaluate the intermediate role of ROS, desiccation, and radiation in influencing the abundance, diversity, and evolved capabilities of Rr bacteria. An antioxidant system of Rr bacteria must be carefully studied to investigate its role in cancer therapy, lung disorders, and the importance of bioremediation of radioactive waste.

## 8 Conclusion

The deserts of the world are rich in Rr and Dr. bacteria. Deserts are mostly desiccated and irradiated. These ecological factors are evidence of the rich diversity of radio-tolerant bacteria. The higher resistance against radiation, desiccation, and oxidation is more evident in the link between ROS species. ROS plays a central role in the origin of a common defensive system, such as an antioxidant system of bacteria against radiation and desiccation. Deserts are home to ROS due to photodesiccated soil. These ROS are evident in the adaptation of bacterial communities in deserts. The antioxidant system regulates cellular stresses by scavenging free radicals among the bacterial community, which inhibits them in deserts. A rise of ROS in the cell damages cellular components and breaks DNA molecules, causing protein oxidation, lipid peroxidation, and abnormality in metabolism. ROS species are mediums that link the toxicity of desiccation to radiation. ROS induction through these stresses also activates counter-responses, which include DNA repair enzymes, an efficient proteomic system, distinct pigmentation, and metabolic and anti-scavenging pathways that reduce toxicity to cellular contents. The similar targets of radiation and desiccation through ROS and the common protection mechanism of Rr bacteria to combat these stresses explained their coevolution. The Rr bacteria in deserts tend to possess more enzymes and extremolytes of biotechnological importance. Due to their resistant nature, Rr bacteria could be efficiently utilized as an expression system in gene cloning. *E. coli* and *Saccharomyces cerevisiae* are platforms of choice for genetic engineering and biotechnological applications. However, in recent years, due to the specific ability to grow and express novel engineered functions, research has begun using Rr bacteria in biotechnologies and bioremediation. Rr bacterial species appear to have a distinct pigmented appearance. Most bacterial species bear blue, red, green, and yellow appearances, and researchers have studied them as reliable sources of natural colorants. Furthermore, more microbial extracts of these species are tested for their antioxidant ability in biomedical research (Sajjad et al., 2020). These bacterial species absorb incoming radiations, dissipate a certain amount in the form of heat, and remain transformed into photoelectric, electronic, and chemical based on their consumption and cellular needs. Rr bacteria is worth exploring due to its importance in pharmaceutical companies, radioactive site decontamination, industrial economic interest, genetic engineering, and biotechnological products.

## Author contributions

AK: Conceptualization, Visualization, Writing – original draft. GL: Formal analysis, Validation, Writing – review & editing. GZ: Conceptualization, Funding acquisition, Supervision, Validation, Writing – review & editing. XL: Formal analysis, Funding acquisition, Supervision, Validation, Visualization, Writing – review & editing.

## References

[ref1] Asker D. Awad T. S. Beppu T. Ueda K. (2012). Isolation, characterization, and diversity of novel radiotolerant carotenoid-producing bacteria. Microb. Caroten. Bact. Microalgae Methods Protoc. 892, 21–60. doi: 10.1007/978-1-61779-879-5_3, PMID: 22623296

[ref2] Atri D. Kamenetskiy M. May M. Kalra A. Castelblanco A. Quiñones-Camacho A. (2022). Estimating the potential of ionizing radiation-induced radiolysis for microbial metabolism in terrestrial planets with rarefied atmospheres arXiv preprint arXiv:2207.14675.

[ref3] Austin A. T. Vivanco L. (2006). Plant litter decomposition in a semi-arid ecosystem controlled by photodegradation. Nature 442, 555–558. doi: 10.1038/nature05038, PMID: 16885982

[ref4] Azua-Bustos A. Urrejola C. Vicuña R. (2012). Life at the dry edge: microorganisms of the Atacama Desert. FEBS Lett. 586, 2939–2945. doi: 10.1016/j.febslet.2012.07.025, PMID: 22819826

[ref5] Bagwell C. Milliken C. Ghoshroy S. Blom D. (2008). Intracellular copper accumulation enhances the growth of *Kineococcus radiotolerans* during chronic irradiation. Appl. Environ. Microbiol. 74, 1376–1384. doi: 10.1128/AEM.02175-07, PMID: 18192425 PMC2258627

[ref6] Basu B. (2022). The radiophiles of Deinococcaceae family: resourceful microbes for innovative biotechnological applications. Curr. Res. Microb. Sci. 3:100153. doi: 10.1016/j.crmicr.2022.100153 35909625 PMC9325910

[ref7] Battista J. R. (1997). Against all odds: the survival strategies of *Deinococcus radiodurans*. Ann. Rev. Microbiol. 51, 203–224. doi: 10.1146/annurev.micro.51.1.203, PMID: 9343349

[ref8] Beblo-Vranesevic K. Galinski E. A. Rachel R. Huber H. Rettberg P. (2017). Influence of osmotic stress on desiccation and irradiation tolerance of (hyper)-thermophilic microorganisms. Arch. Microbiol. 199, 17–28. doi: 10.1007/s00203-016-1269-6, PMID: 27443666

[ref9] Belov A. A. Cheptsov V. S. Vorobyova E. A. (2018). Soil bacterial communities of Sahara and Gibson deserts: physiological and taxonomical characteristics. AIMS Microbiol. 4, 685–710. doi: 10.3934/microbiol.2018.4.685, PMID: 31294242 PMC6613332

[ref10] Buenger J. Driller H. (2004). Ectoin: an effective natural substance to prevent UVA-induced premature photoaging. Skin Pharmacol. Appl. Ski. Physiol. 17, 232–237. doi: 10.1159/000080216, PMID: 15452409

[ref11] Buommino E. Schiraldi C. Baroni A. Paoletti I. Lamberti M. De Rosa M.. (2005). Ectoine from halophilic microorganisms induces the expression of hsp70 and hsp70B′ in human keratinocytes modulating the proinflammatory response. Cell Stress Chaperones 10, 197–203. doi: 10.1379/CSC-101R.1, PMID: 16184764 PMC1226017

[ref12] Burda K. (2014). Potential role of carotenoids as antioksidants in human healihand disease. Nutrients 6, 466–488. doi: 10.3390/nu6020466 24473231 PMC3942711

[ref13] Castillo H. Schoderbek D. Dulal S. Escobar G. Wood J. Nelson R.. (2015). Stress induction in the bacteria Shewanella oneidensis and *Deinococcus radiodurans* in response to below-background ionizing radiation. Int. J. Radiat. Biol. 91, 749–756. doi: 10.3109/09553002.2015.1062571, PMID: 26073528

[ref14] Castillo H. Smith G. B. (2017). Below-background ionizing radiation as an environmental cue for bacteria. Front. Microbiol. 8:177. doi: 10.3389/fmicb.2017.00177

[ref15] Chanal A. Chapon V. Benzerara K. Barakat M. Christen R. Achouak W.. (2006). The desert of Tataouine: an extreme environment that hosts a wide diversity of microorganisms and radiotolerant bacteria. Environ. Microbiol. 8, 514–525. doi: 10.1111/j.1462-2920.2005.00921.x, PMID: 16478457

[ref16] Chandra R. Venkata Mohan S. Roberto P.-S. Ritmann B. E. Cornejo R. A. S. (2018). Biophotovoltaics: Conversion of light energy to bioelectricity through photosynthetic microbial fuel cell technology. Microbial Fuel Cell: A Bioelectrochemical System that Converts Waste to Watts, 373–387. doi: 10.1007/978-3-319-66793-5_19

[ref17] Chaudhary N. Kumar G. (2023). Mutagenic radiations: X-rays, ionizing particles, and ultraviolet. Biotechnologies and genetics in plant mutation breeding: 1: mutagenesis and crop improvement.

[ref18] Chen A. Hernandez-Vargas J. Han R. Cortazar-Martinez O. Gonzalez N. Patel S.. (2021). Small RNAs as a new platform for tuning the biosynthesis of silver nanoparticles for enhanced material and functional properties. ACS Appl. Mater. Interfaces 13, 36769–36783. doi: 10.1021/acsami.1c07400 34319072

[ref19] Cox M. M. Battista J. R. (2005). *Deinococcus radiodurans*—the consummate survivor. Nat. Rev. Microbiol. 3, 882–892. doi: 10.1038/nrmicro1264, PMID: 16261171

[ref20] Daly M. J. (2006). Modulating radiation resistance: insights based on defenses against reactive oxygen species in the radioresistant bacterium *Deinococcus radiodurans*. Clin. Lab. Med. 26, 491–504. doi: 10.1016/j.cll.2006.03.009, PMID: 16815462

[ref21] Daly M. J. (2012). Death by protein damage in irradiated cells. DNA Repair 11, 12–21. doi: 10.1016/j.dnarep.2011.10.024 22112864

[ref22] Daly M. J. Gaidamakova E. K. Matrosova V. Y. Kiang J. G. Fukumoto R. Lee D.-Y.. (2010). Small-molecule antioxidant proteome-shields in *Deinococcus radiodurans*. PLoS One 5:e12570. doi: 10.1371/journal.pone.0012570, PMID: 20838443 PMC2933237

[ref23] Dartnell L. R. Hunter S. J. Lovell K. V. Coates A. J. Ward J. M. (2010). Low-temperature ionizing radiation resistance of Deinococcus radiodurans and Antarctic Dry Valley bacteria. Astrobiology 10, 717–732. doi: 10.1089/ast.2009.0439, PMID: 20950171

[ref24] de Cárcer G. Perez de Castro I. Malumbres M. (2007). Targeting cell cycle kinases for cancer therapy. Curr. Med. Chem. 14, 969–985. doi: 10.2174/092986707780362925 17439397

[ref25] De Groot A. Chapon V. Servant P. Christen R. Saux M. F.-L. Sommer S.. (2005). *Deinococcus deserti* sp. nov., a gamma-radiation-tolerant bacterium isolated from the Sahara Desert. Int. J. Syst. Evol. Microbiol. 55, 2441–2446. doi: 10.1099/ijs.0.63717-0 16280508

[ref26] De la Coba F. Aguilera J. De Galvez M. Alvarez M. Gallego E. Figueroa F.. (2009). Prevention of the ultraviolet effects on clinical and histopathological changes, as well as the heat shock protein-70 expression in mouse skin by topical application of algal UV-absorbing compounds. J. Dermatol. Sci. 55, 161–169. doi: 10.1016/j.jdermsci.2009.06.004, PMID: 19586754

[ref27] Duan Z. Ji D. Weinstein E. J. Liu X. Susa M. Choy E.. (2010). Lentiviral shRNA screen of human kinases identifies PLK1 as a potential therapeutic target for osteosarcoma. Cancer Lett. 293, 220–229. doi: 10.1016/j.canlet.2010.01.014, PMID: 20144850

[ref28] Etemadifar Z. Gholami M. Derikvand P. (2016). UV-resistant bacteria with multiple-stress tolerance isolated from desert areas in Iran. Geomicrobiol J. 33, 1–7. doi: 10.1080/01490451.2015.1063025

[ref29] Fagliarone C. Mosca C. Ubaldi I. Verseux C. Baqué M. Wilmotte A.. (2017). Avoidance of protein oxidation correlates with the desiccation and radiation resistance of hot and cold desert strains of the cyanobacterium Chroococcidiopsis. Extremophiles 21, 981–991. doi: 10.1007/s00792-017-0957-8, PMID: 28856526

[ref30] Fernández Zenoff V. Siñeriz F. Farias M. E. (2006). Diverse responses to UV-B radiation and repair mechanisms of bacteria isolated from high-altitude aquatic environments. Appl. Environ. Microbiol. 72, 7857–7863. doi: 10.1128/AEM.01333-06, PMID: 17056692 PMC1694205

[ref31] Gabani P. Singh O. V. (2013). Radiation-resistant extremophiles and their potential in biotechnology and therapeutics. Appl. Microbiol. Biotechnol. 97, 993–1004. doi: 10.1007/s00253-012-4642-7, PMID: 23271672

[ref32] Galeriu D. Davis P. Raskob W. Melintescu A. (2008). Recent progresses in tritium radioecology and dosimetry. Fusion Sci. Technol. 54, 237–242. doi: 10.13182/FST08-A1803

[ref33] Georgiou C. D. Sun H. J. McKay C. P. Grintzalis K. Papapostolou I. Zisimopoulos D.. (2015). Evidence for photochemical production of reactive oxygen species in desert soils. Nat. Commun. 6:7100. doi: 10.1038/ncomms8100, PMID: 25960012

[ref34] Griffiths D. (2020). Introduction to elementary particles: John Wiley & Sons.

[ref35] Guan N. Li J. Shin H.-D. Du G. Chen J. Liu L. (2017). Microbial response to environmental stresses: from fundamental mechanisms to practical applications. Appl. Microbiol. Biotechnol. 101, 3991–4008. doi: 10.1007/s00253-017-8264-y, PMID: 28409384

[ref36] Guesmi S. Najjari A. Pujic P. Ghedira K. Ouertani R. Jabberi M.. (2022). Roots of the xerophyte Panicum turgidum host a cohort of ionizing-radiation-resistant biotechnologically-valuable bacteria. Saudi J. Biolog. Sci. 29, 1260–1268. doi: 10.1016/j.sjbs.2021.09.020, PMID: 35197792 PMC8847929

[ref37] Guesmi S. Pujic P. Nouioui I. Dubost A. Najjari A. Ghedira K.. (2021). Ionizing-radiation-resistant *Kocuria rhizophila* PT10 isolated from the Tunisian Sahara xerophyte Panicum turgidum: Polyphasic characterization and proteogenomic arsenal. Genomics 113, 317–330. doi: 10.1016/j.ygeno.2020.11.029, PMID: 33279651

[ref38] Halliwell B. Gutteridge J. M. (2015). Free radicals in biology and medicine. Oxford: Oxford University Press.

[ref39] Han R. Fang J. Jiang J. Gaidamakova E. K. Tkavc R. Daly M. J.. (2020). Signal recognition particle RNA contributes to oxidative stress response in *Deinococcus radiodurans* by modulating catalase localization. Front. Microbiol. 11:613571. doi: 10.3389/fmicb.2020.613571, PMID: 33391243 PMC7775534

[ref40] Harris D. R. Tanaka M. Saveliev S. V. Jolivet E. Earl A. M. Cox M. M.. (2004). Preserving genome integrity: the DdrA protein of *Deinococcus radiodurans* R1. PLoS Biol. 2:e304. doi: 10.1371/journal.pbio.0020304, PMID: 15361932 PMC515370

[ref41] He X. Xue J. Shi L. Kong Y. Zhan Q. Sun Y.. (2022). Recent antioxidative nanomaterials toward wound dressing and disease treatment via ROS scavenging. Mater. Today Nano 17:100149. doi: 10.1016/j.mtnano.2021.100149

[ref42] Hezbri K. Ghodhbane-Gtari F. Montero-Calasanz M. D. C. Nouioui I. Rohde M. Spröer C.. (2016). Geodermatophilus pulveris sp. nov., a gamma-radiation-resistant actinobacterium isolated from the Sahara desert. Int. J. Syst. Evol. Microbiol. 66, 3828–3834. doi: 10.1099/ijsem.0.001272, PMID: 27381197

[ref43] Hussain F. Khan I. U. Habib N. Xian W.-D. Hozzein W. N. Zhang Z.-D.. (2016). Deinococcus saudiensis sp. nov., isolated from desert. Int. J. Syst. Evol. Microbiol. 66, 5106–5111. doi: 10.1099/ijsem.0.001479, PMID: 27600000

[ref44] Imlay J. A. (2013). The molecular mechanisms and physiological consequences of oxidative stress: lessons from a model bacterium. Nat. Rev. Microbiol. 11, 443–454. doi: 10.1038/nrmicro3032, PMID: 23712352 PMC4018742

[ref45] Imlay J. A. (2015). Diagnosing oxidative stress in bacteria: not as easy as you might think. Curr. Opin. Microbiol. 24, 124–131. doi: 10.1016/j.mib.2015.01.004, PMID: 25666086 PMC4380616

[ref46] Ito T. Kato M. Toi K. Shirakawa T. Ikemoto I. Tokuda T. (1985). Oxygen species adsorbed on ultraviolet-irradiated magnesium oxide. J. Chem. Soc. Faraday Trans. Phys. Chem. Condens. Phases 81, 2835–2844. doi: 10.1039/f19858102835

[ref9001] Jean-Baptiste P. Fontugne M. Fourré E. Marang L. Antonelli C. Charmasson S.. (2018). Tritium and radiocarbon levels in the Rhône river delta and along the French Mediterranean coastline. J. Environ. Radioact. 187, 53–64. 29433756 10.1016/j.jenvrad.2018.01.031

[ref47] Jiang L.-L. Zhou J.-J. Quan C.-S. Xiu Z.-L. (2017). Advances in industrial microbiome based on microbial consortium for biorefinery. Bioresour. Bioprocess. 4, 1–10. doi: 10.1186/s40643-017-0141-0 28251041 PMC5306255

[ref48] Kanekar P. P. Kanekar S. P. (2022). Psychrophilic, Psychrotrophic, and Psychrotolerant microorganisms diversity and biotechnology of Extremophilic microorganisms from India. Divers. Biotechnol. Extremop. Microorgan. India Microorgan. Sustain., 215–249. doi: 10.1007/978-981-19-1573-4_7

[ref49] Khan A. Naeem M. Bilal M. Khan A. Subhan F. Ikram M.. (2021). Assessing the physico-chemical parameters and some metals of underground water and associated soil in the arid and semiarid regions of Tank District, Khyber Pakhtunkhwa, Pakistan. Environ. Monit. Assess. 193:610. doi: 10.1007/s10661-021-09370-x, PMID: 34462828

[ref50] Kramer D. M. Evans J. R. (2011). The importance of energy balance in improving photosynthetic productivity. Plant Physiol. 155, 70–78. doi: 10.1104/pp.110.166652 21078862 PMC3075755

[ref51] Krasin F. Hutchinson F. (1977). Repair of DNA double-strand breaks in *Escherichia coli*, which requires recA function and the presence of a duplicate genome. J. Mol. Biol. 116, 81–98. doi: 10.1016/0022-2836(77)90120-6, PMID: 338918

[ref52] Kreusch M. G. Duarte R. T. D. (2021). Photoprotective compounds and radioresistance in pigmented and non-pigmented yeasts. Appl. Microbiol. Biotechnol. 105, 3521–3532. doi: 10.1007/s00253-021-11271-5, PMID: 33900423

[ref53] Krinsky N. I. Johnson E. J. (2005). Carotenoid actions and their relation to health and disease. Mol. Asp. Med. 26, 459–516. doi: 10.1016/j.mam.2005.10.001 16309738

[ref54] Krisko A. Radman M. (2010). Protein damage and death by radiation in Escherichia coli and *Deinococcus radiodurans*. Proc. Natl. Acad. Sci. U.S.A. 107, 14373–14377. doi: 10.1073/pnas.1009312107 20660760 PMC2922536

[ref55] Krisko A. Radman M. (2013). Biology of extreme radiation resistance: the way of *Deinococcus radiodurans*. Cold Spring Harb. Perspect. Biol. 5:a012765. doi: 10.1101/cshperspect.a012765 23818498 PMC3685888

[ref56] Kumar V. Kumari A. Pandey M. Sharma M. (2022). Molecular mechanism of radio-resistance and heavy metal tolerance adaptation in microbes. Microb. Extremoz. B978, 275–293. doi: 10.1016/B978-0-12-822945-3.00003-8

[ref57] Kumar R. Patel D. D. Bansal D. D. Mishra S. Mohammed A. Arora R.. (2010). Extremophiles: sustainable resource of natural compounds-extremolytes. Sustain. Biotechnol. Sourc. Renew. Ener. 3295, 279–294. doi: 10.1007/978-90-481-3295-9_15

[ref58] Latifi A. Ruiz M. Zhang C.-C. (2009). Oxidative stress in cyanobacteria. FEMS Microbiol. Rev. 33, 258–278. doi: 10.1111/j.1574-6976.2008.00134.x 18834454

[ref59] Leibel S. Post M. (2016). Endogenous and exogenous stem/progenitor cells in the lung and their role in the pathogenesis and treatment of pediatric lung disease. Front. Pediatr. 4:36. doi: 10.3389/fped.2016.00036 27148506 PMC4830813

[ref60] Leszczynski D. (2014). Radiation proteomics: a brief overview. Proteomics 14, 481–488. doi: 10.1002/pmic.201300390, PMID: 24376023

[ref61] Li J. Gao R. Chen Y. Xue D. Han J. Wang J.. (2020). Isolation and identification of Microvirga thermotolerans HR1, a novel thermo-tolerant bacterium, and comparative genomics among Microvirga species. Microorganisms 8:101. doi: 10.3390/microorganisms8010101, PMID: 31936875 PMC7022394

[ref62] Li Z.-Y. Yang Y. Ming M. Liu B. (2011). Mitochondrial ROS generation for regulation of autophagic pathways in cancer. Biochem. Biophys. Res. Commun. 414, 5–8. doi: 10.1016/j.bbrc.2011.09.046 21951851

[ref9002] Liger K. Grisolia C. Cristescu I. Moreno C. Malard V. Coombs D.. (2018). Overview of the TRANSAT (TRANSversal Actions for Tritium) project. Fusion Eng. Des. 136, 168–172.

[ref63] Liu Y. Chen T. Li J. Wu M. Liu G. Zhang W.. (2022). High proportions of radiation-resistant strains in Culturable bacteria from the Taklimakan Desert. Biology 11:501. doi: 10.3390/biology11040501, PMID: 35453702 PMC9030528

[ref64] Liu M. Dai J. Liu Y. Cai F. Wang Y. Rahman E.. (2011). *Desertibacter roseus* gen. Nov., sp. nov., a gamma radiation-resistant bacterium in the family Rhodospirillaceae, isolated from desert sand. Int. J. Syst. Evol. Microbiol. 61, 1109–1113. doi: 10.1099/ijs.0.021246-0 20543156

[ref65] Liu Z. Kim M. C. Wang L. Zhu G. Zhang Y. Huang Y.. (2017). Deinococcus taklimakanensis sp. nov., isolated from desert soil. Int. J. Syst. Evol. Microbiol. 67, 4311–4316. doi: 10.1099/ijsem.0.002168, PMID: 28984562

[ref66] Llewellyn C. A. Airs R. L. (2010). Distribution and abundance of MAAs in 33 species of microalgae across 13 classes. Mar. Drugs 8, 1273–1291. doi: 10.3390/md8041273, PMID: 20479978 PMC2866486

[ref67] Luo X. Zeng X.-C. He Z. Lu X. Yuan J. Shi J.. (2014). Isolation and characterization of a radiation-resistant bacterium from Taklamakan Desert showing potent ability to accumulate Lead (II) and considerable potential for bioremediation of radioactive wastes. Ecotoxicology 23, 1915–1921. doi: 10.1007/s10646-014-1325-4 25182517

[ref68] Ma R. Zhang Y. Hong H. Lu W. Lin M. Chen M.. (2011). Improved osmotic tolerance and ethanol production of ethanologenic *Escherichia coli* by IrrE, a global regulator of radiation-resistance of *Deinococcus radiodurans*. Curr. Microbiol. 62, 659–664. doi: 10.1007/s00284-010-9759-2, PMID: 20959988

[ref69] Makarova K. S. Aravind L. Wolf Y. I. Tatusov R. L. Minton K. W. Koonin E. V.. (2001). Genome of the extremely radiation-resistant bacterium *Deinococcus radiodurans* viewed from the perspective of comparative genomics. Microbiol. Mol. Biol. Rev. 65, 44–79. doi: 10.1128/MMBR.65.1.44-79.2001, PMID: 11238985 PMC99018

[ref70] Makarova K. S. Omelchenko M. V. Gaidamakova E. K. Matrosova V. Y. Vasilenko A. Zhai M.. (2007). *Deinococcus geothermalis*: the pool of extreme radiation resistance genes shrinks. PLoS One 2:e955. doi: 10.1371/journal.pone.0000955, PMID: 17895995 PMC1978522

[ref71] Makhneva Z. Ashikhmin A. Bolshakov M. Moskalenko A. (2020). Carotenoids are probably involved in singlet oxygen generation in the membranes of purple photosynthetic bacteria under light irradiation. Microbiology 89, 164–173. doi: 10.1134/S0026261720010099

[ref72] Manobala T. Shukla S. K. Subba Rao T. Dharmendira Kumar M. (2019). A new uranium bioremediation approach using radio-tolerant *Deinococcus radiodurans* biofilm. J. Biosci. 44, 1–9. doi: 10.1007/s12038-019-9942-y 31719231

[ref73] Maqbool I. Sudharsan M. Kanimozhi G. Alrashood S. T. Khan H. A. Prasad N. R. (2020). Crude cell-free extract from *Deinococcus radiodurans* exhibit anticancer activity by inducing apoptosis in triple-negative breast cancer cells. Front. Cell Dev. Biol. 8:707. doi: 10.3389/fcell.2020.00707, PMID: 32850827 PMC7409529

[ref74] Markillie L. M. Varnum S. M. Hradecky P. Wong K.-K. (1999). Targeted mutagenesis by duplication insertion in the radioresistant bacterium *Deinococcus radiodurans*: radiation sensitivities of catalase (katA) and superoxide dismutase (sodA) mutants. J. Bacteriol. 181, 666–669. doi: 10.1128/JB.181.2.666-669.1999, PMID: 9882685 PMC93425

[ref75] Maróti P. Kovács I. A. Kis M. Smart J. L. Iglói F. (2020). Correlated clusters of closed reaction centers during induction of intact cells of photosynthetic bacteria. Sci. Rep. 10:14012. doi: 10.1038/s41598-020-70966-3, PMID: 32814810 PMC7438532

[ref76] Marszalkowski M. Werner A. Feltens R. Helmecke D. Gößringer M. Westhof E.. (2021). Comparative study on tertiary contacts and folding of RNase P RNAs from a psychrophilic, a mesophilic/radiation-resistant, and a thermophilic bacterium. RNA 27, 1204–1219. doi: 10.1261/rna.078735.121, PMID: 34266994 PMC8457005

[ref77] Mba C. Afza R. Shu Q. (2012). Mutagenic radiations: X-rays, ionizing particles and ultraviolet plant mutation breeding and biotechnology. Wallingford UK: CABI.

[ref78] McCalley C. K. Sparks J. P. (2009). Abiotic gas formation drives nitrogen loss from a desert ecosystem. Science 326, 837–840. doi: 10.1126/science.1178984, PMID: 19892980

[ref79] McLean R. McLean M. (2010). Microbial survival mechanisms and the interplanetary transfer of life through space. J. Cosmol. 7, 1802–1820.

[ref80] Melintescu A. Galeriu D. (2011). Dynamic model for tritium transfer in an aquatic food chain. Radiat. Environ. Biophys. 50, 459–473. doi: 10.1007/s00411-011-0362-0, PMID: 21499903

[ref81] Mendes-Silva T. D. C. D. da Silva Andrade R. F. Ootani M. A. Mendes P. V. D. Da Silva M. R. F. Souza K. S.. (2020). Biotechnological potential of carotenoids produced by extremophilic microorganisms and application prospects for the cosmetics industry. Adv. Microbiol. 10, 397–410. doi: 10.4236/aim.2020.108029

[ref82] Misra C. S. Appukuttan D. Kantamreddi V. S. S. Rao A. S. Apte S. K. (2012). *Recombinant D. radiodurans* cells for bioremediation of heavy metals from acidic/neutral aqueous wastes. Bioengineered 3, 44–48. doi: 10.4161/bbug.3.1.18878, PMID: 22179144

[ref83] Mitchell D. L. Karentz D. (1993). The induction and repair of DNA photodamage in the environment. Environ. UV Photobiol., 345–377. doi: 10.1007/978-1-4899-2406-3_12

[ref84] Mohseni M. Abbaszadeh J. Nasrollahi Omran A. (2014). Radiation resistant of native Deinococcus spp. isolated from the lout desert of Iran “the hottest place on earth”. Int. J. Environ. Sci. Technol. 11, 1939–1946. doi: 10.1007/s13762-014-0643-7

[ref85] Mortimer R. K. (1958). Radiobiological and genetic studies on a polyploid series (haploid to hexaploid) of *Saccharomyces cerevisiae*. Radiat. Res. 9, 312–326. doi: 10.2307/3570795, PMID: 13579200

[ref86] Murugan R. N. Park J.-E. Kim E.-H. Shin S. Y. Cheong C. Lee K. S.. (2011). Plk1-targeted small molecule inhibitors: molecular basis for their potency and specificity. Mol. Cells 32, 209–220. doi: 10.1007/s10059-011-0126-3, PMID: 21809214 PMC3887635

[ref87] Musilova M. Wright G. Ward J. M. Dartnell L. R. (2015). Isolation of radiation-resistant bacteria from Mars analog Antarctic dry valleys by preselection, and the correlation between radiation and desiccation resistance. Astrobiology 15, 1076–1090. doi: 10.1089/ast.2014.1278, PMID: 26684506 PMC4683558

[ref88] Nayak T. Sengupta I. Dhal P. K. (2021). A new era of radiation resistance bacteria in bioremediation and production of bioactive compounds with therapeutic potential and other aspects: An in-perspective review. J. Environ. Radioact. 237:106696. doi: 10.1016/j.jenvrad.2021.106696, PMID: 34265519

[ref9003] Oms P. E. Du Bois P. B. Dumas F. Lazure P. Morillon M. Voiseux C.. (2019). Inventory and distribution of tritium in the oceans in 2016. Sci. Total Environ. 656, 1289–1303. 30625658 10.1016/j.scitotenv.2018.11.448

[ref89] Orellana G. Gómez-Silva B. Urrutia M. Galetović A. (2020). UV-A irradiation increases scytonemin biosynthesis in cyanobacteria inhabiting halites at Salar Grande, Atacama Desert. Microorganisms 8:1690. doi: 10.3390/microorganisms8111690, PMID: 33142998 PMC7692114

[ref90] Pan J. Wang J. Zhou Z. Yan Y. Zhang W. Lu W.. (2009). IrrE, a global regulator of extreme radiation resistance in *Deinococcus radiodurans*, enhances salt tolerance in Escherichia coli and *Brassica napus*. PLoS One 4:e4422. doi: 10.1371/journal.pone.0004422, PMID: 19204796 PMC2635966

[ref91] Paulino-Lima I. G. Azua-Bustos A. Vicuña R. González-Silva C. Salas L. Teixeira L.. (2013). Isolation of UVC-tolerant bacteria from the hyperarid Atacama Desert, Chile. Microb. Ecol. 65, 325–335. doi: 10.1007/s00248-012-0121-z, PMID: 23001596

[ref92] Peana M. Chasapis C. Simula G. Medici S. Zoroddu M. (2018). A model for manganese interaction with *Deinococcus radiodurans* proteome network involved in ROS response and defense. J. Trace Elem. Med. Biol. 50, 465–473. doi: 10.1016/j.jtemb.2018.02.001, PMID: 29449107

[ref93] Peng F. Zhang L. Luo X. Dai J. An H. Tang Y.. (2009). *Deinococcus xinjiangensis* sp. nov., isolated from desert soil. Int. J. Syst. Evol. Microbiol. 59, 709–713. doi: 10.1099/ijs.0.004564-0, PMID: 19329593

[ref94] Qi H.-Z. Wang W.-Z. He J.-Y. Ma Y. Xiao F.-Z. He S.-Y. (2020). Antioxidative system of *Deinococcus radiodurans*. Res. Microbiol. 171, 45–54. doi: 10.1016/j.resmic.2019.11.002 31756434

[ref95] Rai S. N. Dutta T. (2024). A novel ionizing radiation-induced small RNA, DrsS, promotes the detoxification of reactive oxygen species in *Deinococcus radiodurans*. Appl. Environ. Microbiol. 90, e01538–e01523. doi: 10.1128/aem.01538-23 38587394 PMC11107164

[ref96] Rainey F. A. Ferreira M. Nobre M. F. Ray K. Bagaley D. Earl A. M.. (2007). *Deinococcus peraridilitoris* sp. nov., isolated from a coastal desert. Int. J. Syst. Evol. Microbiol. 57, 1408–1412. doi: 10.1099/ijs.0.64956-0, PMID: 17625166

[ref97] Rainey F. A. Ray K. Ferreira M. Gatz B. Z. Nobre M. F. Bagaley D.. (2005). Extensive diversity of ionizing-radiation-resistant bacteria recovered from Sonoran Desert soil and description of nine new species of the genus Deinococcus obtained from a single soil sample. Appl. Environ. Microbiol. 71, 5225–5235. doi: 10.1128/AEM.71.9.5225-5235.2005, PMID: 16151108 PMC1214641

[ref98] Ranawat P. Rawat S. (2017). Radiation resistance in thermophiles: mechanisms and applications. World J. Microbiol. Biotechnol. 33, 1–22. doi: 10.1007/s11274-017-2279-5 28470425

[ref99] Redza-Dutordoir M. Averill-Bates D. (2016). Biochim Biophys Acta-Mol. Cell Res. 1863, 2977–2992. 10.1016/j.bbamcr.2016.09.012 27646922

[ref100] Renger G. (ed.). (2007). Primary processes of photosynthesis: Principles and apparatus. (Vol. 8). Royal Society of Chemistry.

[ref101] Rivera-Madrid R. Carballo-Uicab V. M. Cárdenas-Conejo Y. Aguilar-Espinosa M. Siva R. (2020). Overview of carotenoids and beneficial effects on human health. Carotenoids Proper. Process. Appl., 1–40.

[ref102] Roch-Lefèvre S. Grégoire E. Martin-Bodiot C. Flegal M. Fréneau A. Blimkie M.. (2018). Cytogenetic damage analysis in mice chronically exposed to low-dose internal tritium beta-particle radiation. Oncotarget 9, 27397–27411. doi: 10.18632/oncotarget.25282, PMID: 29937993 PMC6007944

[ref103] Saito T. (2011). The red pigments contained in the radioresistant bacteria and the radioresistant mechanism of these bacteria.

[ref104] Sajjad W. Ahmad M. Khan S. Ilyas S. Hasan F. Celik C.. (2017). Radio-protective and antioxidative activities of astaxanthin from newly isolated radio-resistant bacterium Deinococcus sp. strain WMA-LM9. Ann. Microbiol. 67, 443–455. doi: 10.1007/s13213-017-1269-z

[ref105] Sajjad W. Din G. Rafiq M. Iqbal A. Khan S. Zada S.. (2020). Pigment production by cold-adapted bacteria and fungi: colorful tale of cryosphere with wide range applications. Extremophiles 24, 447–473. doi: 10.1007/s00792-020-01180-2, PMID: 32488508 PMC7266124

[ref106] Sajjad W. Khan S. Ahmad M. Rafiq M. Badshah M. Zada S.. (2018). Effects of ultra-violet radiation on cellular proteins and lipids of radioresistant bacteria isolated from desert soil. Folia Biologica (Kraków) 66, 41–52. doi: 10.3409/fb_66-1.05

[ref107] Santos A. L. Gomes N. C. Henriques I. Almeida A. Correia A. Cunha A. (2013). Role of transition metals in UV-B-induced damage to Bacteria. Photochem. Photobiol. 89, 640–648. doi: 10.1111/php.12049 23360113

[ref108] Schieber M. Chandel N. S. (2014). ROS function in redox signaling and oxidative stress. Curr. Biol. 24, R453–R462. doi: 10.1016/j.cub.2014.03.034, PMID: 24845678 PMC4055301

[ref109] Schneider J. Matsuoka M. Takeuchi M. Zhang J. Horiuchi Y. Anpo M.. (2014). Understanding TiO2 photocatalysis: mechanisms and materials. Chem. Rev. 114, 9919–9986. doi: 10.1021/cr5001892, PMID: 25234429

[ref110] Schulze-Makuch D. Airo A. Schirmack J. (2017). The adaptability of life on earth and the diversity of planetary habitats. Front. Microbiol. 8:2011. doi: 10.3389/fmicb.2017.02011, PMID: 29085352 PMC5650640

[ref111] Sghaier H. (2011). DNA repair: lessons from the evolution of ionizing- radiation-resistant prokaryotes – fact and theory. Select. Top. DNA Repair, 145–156. doi: 10.5772/22312

[ref112] Shirsalimian M. Akhavan Sepahy A. Amoozegar M. Kalantar S. Dabbagh R. (2018). Isolation of two radiation resistant and desiccation tolerant bacteria, Modestobacter sp. A2 and maritalea sp. B9, from gandom beryan hill in the lut desert of Iran. Microbiology 87, 363–371. doi: 10.1134/S0026261718030104

[ref113] Shukla M. Chaturvedi R. Tamhane D. Vyas P. Archana G. Apte S.. (2007). Multiple-stress tolerance of ionizing radiation-resistant bacterial isolates obtained from various habitats: correlation between stresses. Curr. Microbiol. 54, 142–148. doi: 10.1007/s00284-006-0311-3, PMID: 17180747

[ref114] Shukla A. Parmar P. Saraf M. (2017). Radiation, radionuclides and bacteria: An in-perspective review. J. Environ. Radioact. 180, 27–35. doi: 10.1016/j.jenvrad.2017.09.013, PMID: 29024816

[ref115] Shukla S. K. Subba Rao T. (2017). The first recorded incidence of *Deinococcus radiodurans* R1 biofilm formation and its implications in heavy metals bioremediation. BioRxiv 234–781.

[ref116] Singh H. (2018). Desiccation and radiation stress tolerance in cyanobacteria. J. Basic Microbiol. 58, 813–826. doi: 10.1002/jobm.201800216, PMID: 30080267

[ref117] Singh O. V. Gabani P. (2011). Extremophiles: radiation resistance microbial reserves and therapeutic implications. J. Appl. Microbiol. 110, 851–861. doi: 10.1111/j.1365-2672.2011.04971.x, PMID: 21332616

[ref118] Slade D. Radman M. (2011). Oxidative stress resistance in *Deinococcus radiodurans*. Microbiol. Mol. Biol. Rev. 75, 133–191. doi: 10.1128/MMBR.00015-10, PMID: 21372322 PMC3063356

[ref119] Slaninova E. Sedlacek P. Mravec F. Mullerova L. Samek O. Koller M.. (2018). Light scattering on PHA granules protects bacterial cells against the harmful effects of UV radiation. Appl. Microbiol. Biotechnol. 102, 1923–1931. doi: 10.1007/s00253-018-8760-8, PMID: 29349494

[ref120] Stevenson C. S. Capper E. A. Roshak A. K. Marquez B. Eichman C. Jackson J. R.. (2002). The identification and characterization of the marine natural product scytonemin as a novel antiproliferative pharmacophore. J. Pharmacol. Exp. Ther. 303, 858–866. doi: 10.1124/jpet.102.036350, PMID: 12388673

[ref121] Strebhardt K. Ullrich A. (2006). Targeting polo-like kinase 1 for cancer therapy. Nat. Rev. Cancer 6, 321–330. doi: 10.1038/nrc1841 16557283

[ref122] Tanaka M. Earl A. M. Howell H. A. Park M.-J. Eisen J. A. Peterson S. N.. (2004). Analysis of *Deinococcus radiodurans*'s transcriptional response to ionizing radiation and desiccation reveals novel proteins that contribute to extreme radioresistance. Genetics 168, 21–33. doi: 10.1534/genetics.104.029249, PMID: 15454524 PMC1448114

[ref123] Tian B. Sun Z. Shen S. Wang H. Jiao J. Wang L.. (2009). Effects of carotenoids from *Deinococcus radiodurans* on protein oxidation. Lett. Appl. Microbiol. 49, 689–694. doi: 10.1111/j.1472-765X.2009.02727.x 19780959

[ref124] Tian B. Wu Y. Sheng D. Zheng Z. Gao G. Hua Y. (2004). Chemiluminescence assay for reactive oxygen species scavenging activities and inhibition on oxidative damage of DNA in *Deinococcus radiodurans*. Lumin. J. Biol. Chem. Lumin. 19, 78–84. doi: 10.1002/bio.761, PMID: 15098207

[ref125] Tian B. Xu Z. Sun Z. Lin J. Hua Y. (2007). Evaluation of the antioxidant effects of carotenoids from *Deinococcus radiodurans* through targeted mutagenesis, chemiluminescence, and DNA damage analyses. Biochimica et Biophysica Acta (BBA)-General Subjects 1770, 902–911. doi: 10.1016/j.bbagen.2007.01.016, PMID: 17368731

[ref126] Tiquia-Arashiro S. Rodrigues D. F. (2016). Extremophiles: applications in nanotechnology. Springer.

[ref127] Villa J. K. Han R. Tsai C.-H. Chen A. Sweet P. Franco G.. (2021). A small RNA regulates pprM, a modulator of pleiotropic proteins promoting DNA repair, in *Deinococcus radiodurans* under ionizing radiation. Sci. Rep. 11:12949. doi: 10.1038/s41598-021-91335-8, PMID: 34155239 PMC8217566

[ref128] Wang P. Schellhorn H. E. (1995). Induction of resistance to hydrogen peroxide and radiation in *Deinococcus radiodurans*. Can. J. Microbiol. 41, 170–176. doi: 10.1139/m95-023, PMID: 7720013

[ref129] Yang Q. (2021). Crucial roles of carotenoids as bacterial endogenous defense system for bacterial radioresistance of *Deinococcus radiodurans*. BioRxiv 2021:811.

[ref130] Yoboue E. D. Mougeolle A. Kaiser L. Averet N. Rigoulet M. Devin A. (2014). The role of mitochondrial biogenesis and ROS in the control of energy supply in proliferating cells. Biochimica et Biophysica Acta (BBA)-Bioenergetics 1837, 1093–1098. doi: 10.1016/j.bbabio.2014.02.023, PMID: 24602596

[ref131] Yu L. Z. H. Luo X. S. Liu M. Huang Q. (2015). Diversity of ionizing radiation-resistant bacteria obtained from the Taklimakan Desert. J. Basic Microbiol. 55, 135–140. doi: 10.1002/jobm.201300390, PMID: 25590873

[ref132] Yuan M. Zhang W. Dai S. Wu J. Wang Y. Tao T.. (2009). *Deinococcus gobiensis* sp. nov., an extremely radiation-resistant bacterium. Int. J. Syst. Evol. Microbiol. 59, 1513–1517. doi: 10.1099/ijs.0.004523-0, PMID: 19502345

[ref133] Zhang Q. Liu C. Tang Y. Zhou G. Shen P. Fang C.. (2007). *Hymenobacter xinjiangensis* sp. nov., a radiation-resistant bacterium isolated from the desert of Xinjiang, China. Int. J. Syst. Evol. Microbiol. 57, 1752–1756. doi: 10.1099/ijs.0.65033-0 17684250

[ref134] Zhang L. Yang Q. Luo X. Fang C. Zhang Q. Tang Y. (2007). Knockout of crtB or crtI gene blocks the carotenoid biosynthetic pathway in *Deinococcus radiodurans* R1 and influences its resistance to oxidative DNA-damaging agents due to change of free radicals scavenging ability. Arch. Microbiol. 188, 411–419. doi: 10.1007/s00203-007-0262-5, PMID: 17541775

[ref135] Zhao C. Wang G. Zhang M. Wang G. de With G. Bezhenar R.. (2021). Transport and dispersion of tritium from the radioactive water of the Fukushima Daiichi nuclear plant. Mar. Pollut. Bull. 169:112515. doi: 10.1016/j.marpolbul.2021.112515, PMID: 34023585

[ref136] Zimmerman J. M. Battista J. R. (2005). A ring-like nucleoid is not necessary for radioresistance in the Deinococcaceae. BMC Microbiol. 5, 17–10. doi: 10.1186/1471-2180-5-17 15799787 PMC1079854

